# Sociodemographic and Psychological Characteristics of Very Light Smoking Among Women in Emerging Adulthood, National Survey of Drug Use and Health, 2011

**DOI:** 10.5888/pcd12.140547

**Published:** 2015-07-16

**Authors:** Xiaoyin Li, Carole K. Holahan, Charles J. Holahan

**Affiliations:** Author Affiliations: Xiaoyin Li, Charles J. Holahan, University of Texas at Austin, Austin, Texas.

## Abstract

**Introduction:**

Although smoking prevalence and average cigarette consumption have declined, very light smoking (5 or fewer cigarettes per day) has increased. Very light smoking is common among young adult women. This study examines the differences between the sociodemographic and psychosocial factors associated with women in emerging adulthood who are very light smokers and similar women who are at other smoking levels.

**Methods:**

The sample consisted of 9,789 women aged 18 to 25 years who took part in the 2011 National Survey on Drug Use and Health in the United States. Variables were sociodemographic factors, psychological adjustment, substance misuse, smoking attitudes, daily smoking, age at smoking initiation, and nicotine dependence. Analyses used were χ^2^ and multinomial logistic regression.

**Results:**

Almost a fifth of participants and about three-fifths of smokers were very light smokers (no more than 5 cigarettes per day). Very light smokers were relatively more likely than other smokers to be young (aged 18 to 20), to be from a minority group, and to have some college education. They also were less likely to be married. The characteristics of very light smokers (poor psychological adjustment and tendency to misuse other substances) were similar to the characteristics of other smokers. However, very light smokers were more likely than other smokers to recognize high risks in smoking, less likely to report nicotine dependence, and more likely to be nondaily smokers.

**Conclusion:**

Prevention programs targeting women in emerging adulthood need to recognize the prevalence of very light smoking in this population. Although comorbid psychological disorders and substance use present challenges, very light smokers’ perception of higher smoking risks and lower nicotine dependence compared with that of other smokers provide intervention opportunities.

## Introduction

For the past 2 decades, both the prevalence of smoking and average cigarette consumption have declined in the United States (1). However, at the same time, the proportion of very light smoking has increased (1,2). Very light smoking is defined as smoking no more than 5 cigarettes per day (cpd) (3,4). Even a low level of smoking carries significant health risk (5–7). Smoking at very light levels is associated with cardiovascular and pulmonary diseases (6). In addition, very light smoking is associated with increased risk of cancer, particularly lung cancer (5).

Very light smoking is common among women in emerging adulthood and is of global concern (8,9). Emerging adulthood, spanning ages 18 to 25, is an important developmental stage and is the period when the prevalence of most types of risky behaviors is highest (10). In addition to increased risk for cardiovascular disease and lung cancer, cigarette use poses unique health consequences for women (9). Women in emerging adulthood are of reproductive age, and cigarette use before or during pregnancy poses threats to maternal and child health (9,11). Women smokers are at increased risk for delays in conceiving, infertility, and pregnancy-related disorders as well as cancer of the cervix (11).

Key to developing effective interventions targeting very light smoking among women in emerging adulthood is that the interventions be based on an understanding of sociodemographic and psychosocial factors that characterize this subgroup of smokers. Focusing on women in emerging adulthood, the purpose of our study was to examine psychosocial characteristics of very light smokers (≤5 cpd) contrasted with those of never, former, light (6–16 cpd), and heavier (>16 cpd) smokers. On the basis of previous research examining overall cigarette use (1,12), we focused on sociodemographic factors, psychological adjustment, misuse of other substances, attitudes toward smoking, and (among current smokers) daily smoking, age at smoking initiation, and nicotine dependence.

## Methods

Secondary data analyses with the National Survey on Drug Use and Health (NSDUH) data set (13) were conducted to answer the research questions. NSDUH is funded by the Substance Abuse and Mental Health Services Administration (SAMHSA). Since 1971, NSDUH has been conducted annually to collect national data on substance use among individuals aged 12 years or older in the United States. The 2011 NSDUH employed a state-based design with an independent, multistage area probability sample within each state and the District of Columbia, and the questionnaires were administrated by using computer-assisted interviewing. The questionnaire and measurements are reliable and valid (14).

Our sample consisted of 9,789 women aged 18 to 25 years who took part in the 2011 NSDUH survey and who provided valid responses to all selected survey questions. The response rate for the 2011 survey year was 64.7% (14).

### Measures

The question used to define smoking status was “On the one day/days you smoked a cigarette during the past 30 days, how many cigarettes did you smoke per day, on average?” Five categories of smoking status were used: never smokers, former smokers, very light smokers, light smokers, and heavier smokers (3,15), based on responses to the smoking status question. Former smokers were defined as individuals who smoked in the past but have not used cigarettes within the past 30 days; very light smokers were defined as those who are current smokers and smoked 5 cpd or fewer within the past 30 days; light smokers were defined as those who smoked 6 to 16 cpd within the past 30 days; and heavier smokers were defined as those who smoked more than 16 cpd within the past 30 days.

Sociodemographic variables included 2 age groups (18–20 years and 21–25 years); ethnicity (non-Hispanic white, non-Hispanic black, Hispanic, and other ethnicities); marital status (never married; married; widowed, divorced or separated); educational level (less than high school graduate, high school graduate, some college, college graduate); and educational status (enrolled in school, not enrolled in school). Reference groups for the above variables were, respectively, younger than 21, non-Hispanic white, never married, college graduate, and not enrolled in school.

The 6-item K6 screening instrument for nonspecific psychological distress (16) was included to evaluate past month’s psychological distress. Six symptoms addressing frequency of psychological distress during the past 30 days were measured on a scale ranging from 0 to 4, with responses coded with 0 as none of the time, 1 as a little of the time, 2 as some of the time, 3 as most of the time, and 4 as all of the time. Item scores were summed, with total scores ranging from 0 to 24. Total scores less than 13 were coded as not having past month psychological distress (reference group), and total scores 13 or higher were coded as having psychological distress (13).

The NSDUH included questions from the Diagnostic and Statistical Manual (DSM IV) measuring lifetime major depressive symptoms. The questions were adapted from the depression section of the National Comorbidity Survey-Replication (NCS-R) (13). If participants had at least 5 of 9 symptoms of depression (eg, depressed mood or loss of interest or pleasure in daily activities) lasting at least 2 weeks in a year, they were coded as having a lifetime major depressive episode (MDE); if not, they were coded as not having a lifetime MDE (reference group).

Past month binge drinking was defined as drinking 5 or more drinks on the same occasion on at least 1 day in the past 30 days (13). The variable was coded as presence or absence (reference group) of current binge drinking.

A variable indexing any illicit drug use during the past month was created by examining questions on hallucinogens, heroin, marijuana, cocaine, inhalants, and “any psychotherapeutics” (ie, pain relievers, tranquilizers, stimulants, and sedatives) (13). The variable was coded as presence or absence (reference group) of illicit drug use.

A 4-point question, “How much do people risk harming themselves physically and in other ways when they smoke 1 or more packs of cigarettes per day?” was used to determine perceived health risk of smoking among participants (13). Responses were coded with 1 as no risk, 2 as slight risk, 3 as moderate risk, and 4 as great risk.

Daily versus nondaily smoking was assessed by an item that asked respondents “During the past 30 days, on how many days did you smoke part or all of a cigarette?” (13). Nondaily smokers were those who smoked 1 to 29 days in the past 30 days (reference group), and daily smokers were those who smoked every day of the past 30 days. A question asking the age when participants first smoked a cigarette was used to determine their age at smoking initiation.

The 2011 NSDUH contains 17 questions from the Nicotine Dependence Syndrome Scale (NDSS) that address 5 aspects of dependence, including smoking drive, nicotine tolerance, continuous smoking, behavioral priority, and stereotypy. All items were measured on a 5-point scale with values varying from 1 to 5. Item scores were summed to compute subscale scores. Smoking drive was measured by 5 questions about the urge to smoke due to craving and withdrawal symptoms, which are considered essential for addiction. Nicotine tolerance was measured by 3 questions about decreased sensitivity to nicotine or smoking an increasing number of cigarettes since smoking onset. Continuous smoking was measured by 3 items about the constancy of smoking behavior without interruption. Behavioral priority was measured by 2 items about the degree to which smoking was prioritized over other behaviors. Stereotypy was measured by 4 items about the development of day-to-day patterns of smoking and increasing resistance to change.

### Statistical analyses

Initially, we examined sociodemographic factors separately with χ^2^ tests. Then, while controlling for sociodemographic factors, we used multinomial logistic regression to examine differences among the 5 smoking-status groups (very light smokers were the reference group) on psychological adjustment, substance misuse, and perceived health risk of smoking. Finally, among current smokers, while controlling for sociodemographic factors, we used multinomial logistic regression to examine differences among the 3 current-smoking groups (very light smokers were the reference group) on daily smoking, age at smoking initiation, and 5 aspects of nicotine dependence. The .05 level of significance was used throughout. Analyses were conducted using SPSS 21 (IBM Inc). See Berg et al for a similar analytic strategy (15).

## Results

Of the 9,789 participants, 4,069 (41.6%) were never smokers, 2,756 (28.2%) were former smokers, and 2,964 (30.3%) were current smokers. Among the current smokers, 62.4% were very light smokers, 26.7% were light smokers, and 10.8% were heavier smokers. About 71.3% of very light smokers were nondaily smokers (Table 1). Characteristics of participants who were smokers taken from responses to the NDSS are shown in Table 2.

**Table 1:** Characteristics of Women Aged 18 to 25 Years by Smoking Status^a^, National Survey on Drug Use and Health, 2011

Variable	All Participants, N = 9,789	Never Smokers, n = 4,069	Former Smokers, n = 2,756	Very Light Smokers, n = 1,851	Light Smokers, n = 792	Heavier Smokers, n = 321
**Age group, n (%)**						
18–20	3,712 (37.9)	1,797 (44.2)	859 (31.2)	729 (39.4)	239 (30.2)	88 (27.4)
21–25	6,077 (62.1)	2,272 (55.8)	1,897 (68.8)	1,122 (60.6)	553 (69.8)	233 (72.6)
**Ethnicity, n (%)**						
White	5,681 (58.0)	1,983 (48.7)	1,701 (61.7)	1,096 (59.2)	632 (79.8)	269 (83.8)
Black	1,443 (14.7)	854 (21.0)	293 (10.6)	229 (12.4)	48 (6.1)	19 (5.9)
Hispanic	1,735 (17.7)	841 (20.7)	534 (19.4)	306 (16.5)	45 (5.7)	9 (2.8)
Other	930 (9.5)	391 (9.6)	228 (8.3)	220 (11.9)	67 (8.5)	24 (7.5)
**Education level, n (%)**						
College graduate	1,480 (15.1)	686 (16.9)	555 (20.1)	190 (10.3)	37 (4.7)	12 (3.7)
Some college	3,530 (36.1)	1,476 (36.3)	1,093 (39.7)	685 (37.0)	206 (26.0)	70 (21.8)
High school graduate	3,283 (33.5)	1,327 (32.6)	811 (29.4)	665 (35.9)	327 (41.3)	153 (47.7)
Less than high school graduate	1,496 (15.3)	580 (14.3)	297 (10.8)	311 (16.8)	222 (28.0)	86 (26.8)
**Educational status, n (%)**						
Enrolled in school	4,809 (49.1)	2,341 (57.5)	1,326 (48.1)	862 (46.6)	227 (28.7)	53 (16.5)
Not enrolled in school	4,980 (50.9)	1,728 (42.5)	1,430 (51.9)	989 (53.4)	565 (71.3)	268 (83.5)
**Marital status, n (%)**						
Never married	8,054 (82.3)	3,381 (83.1)	2,190 (79.5)	1,622 (87.6)	637 (80.4)	224 (69.8)
Married	1,499 (15.3)	643 (15.8)	503 (18.3)	176 (9.5)	114 (14.4)	63 (19.6)
Widowed, divorced, or separated	236 (2.4)	45 (1.1)	63 (2.3)	53 (2.9)	41 (5.2)	34 (10.6)
**Past month serious psychological distress,^b^ n (%)**						
No	8,875 (90.7)	3,808 (93.6)	2,532 (91.9)	1,609 (86.9)	660 (83.3)	266 (82.9)
Yes	914 (9.3)	261 (6.4)	224 (8.1)	242 (13.1)	132 (16.7)	55 (17.1)
**Lifetime major depressive episode,^c^ n (%)**						
No	7,966 (81.4)	3,532 (86.8)	2,190 (79.5)	1,421 (76.8)	580 (73.2)	243 (75.7)
Yes	1,823 (18.6)	537 (13.2)	566 (20.5)	430 (23.2)	212 (26.8)	78 (24.3)
**Past month binge alcohol use,^d^ n (%)**						
No	6,504 (66.4)	3,364 (82.7)	1,757 (63.8)	821 (44.4)	394 (49.7)	168 (52.3)
Yes	3,285 (33.6)	705 (17.3)	999 (36.2)	1,030 (55.6)	398 (50.3)	153 (47.7)
**Past month illicit drug use,^e^ n (%)**						
No	8,153 (83.3)	3,827 (94.1)	2,348 (85.2)	1,236 (66.8)	523 (66.0)	219 (68.2)
Yes	1,636 (16.7)	242 (5.9)	408 (14.8)	615 (33.2)	269 (34.0)	102 (31.8)
**Perceived risk of smoking,^f^ mean (SD)**	3.60 (0.70)	3.66 (0.66)	3.67 (0.63)	3.52 (0.75)	3.32 (0.78)	3.21 (0.87)

Abbreviation: SD, standard deviation.

a Former smokers were individuals who have smoked in the past but did not do so within the past 30 days; very light smokers were current smokers who smoked 5 or fewer cigarettes per day (cpd) within the past 30 days; light smokers smoked 6 to 16 cpd within the past 30 days; and heavier smokers smoked more than 16 cpd within the past 30 days.

b Six symptoms addressing frequency of psychological distress during the past 30 days were measured on a scale ranging from 0 to 4, with responses coded with 0 as none of the time, 1 as a little of the time, 2 as some of the time, 3 as most of the time, and 4 as all of the time. Item scores were summed, with total scores ranging from 0 to 24. Total scores less than 13 were coded as not having past month psychological distress (reference group), and total scores 13 or higher were coded as having psychological distress.

c If participants had at least 5 of 9 symptoms of depression (eg, depressed mood or loss of interest or pleasure in daily activities) lasting at least 2 weeks in a year, they were coded as having a lifetime major depressive episode.

d Defined as drinking 5 or more drinks on the same occasion on at least 1 day in the past 30 days.

e Presence or absence of illicit drug use, determined from questions on use of hallucinogens, heroin, marijuana, cocaine, inhalants, and “any psychotherapeutics” (ie, pain relievers, tranquilizers, stimulants, and sedatives).

f From responses to the question “How much do people risk harming themselves physically and in other ways when they smoke 1 or more packs of cigarettes per day?” Responses were coded with 1 as no risk, 2 as slight risk, 3 as moderate risk, and 4 as great risk.

**Table 2:** Characteristics of Women Aged 18 to 25 Years Who Are Current Smokers,^a^ National Survey on Drug Use and Health, 2011

Characteristic	Current Smokers	Very Light Smokers	Light Smokers	Heavier Smokers
**Daily smokers, n (%)**				
No	1,436 (48.8)	1,308 (71.3)	98 (12.4)	30 (9.4)
Yes	1,506 (51.2)	527 (28.7)	691 (87.6)	288 (90.6)
**Starting age, mean (SD)**	15.10 (3.04)	15.66 (2.88)	14.24 (2.72)	14.00 (2.95)
**Smoking drive,^b^ mean (SD)**	2.45 (1.13)	1.92 (0.88)	3.27 (0.93)	3.58 (0.91)
**Nicotine tolerance,^c^ mean (SD)**	2.15 (1.18)	1.72 (0.95)	2.74 (1.17)	3.15 (1.16)
**Continuous smoking,^d^ mean (SD)**	3.51 (1.01)	3.70 (1.02)	3.18 (0.88)	3.18 (0.97)
**Behavioral priority,^e^ mean (SD)**	1.35 (0.70)	1.22 (0.54)	1.47 (0.80)	1.83 (0.98)
**Stereotypy,^f^ mean (SD)**	2.47 (1.10)	2.00 (0.93)	3.14 (0.88)	3.50 (0.88)

Abbreviation: SD, standard deviation.

a Current smokers smoked during the past 30 days; very light smokers were current smokers who smoked 5 or fewer cigarettes per day (cpd) within the past 30 days; light smokers smoked 6 to 16 cpd within the past 30 days; and heavier smokers smoked more than 16 cpd within the past 30 days. Maximum number of current smokers was 2,964. A small amount of missing data occurred on daily smoking and the nicotine dependence scales.

b Measured by 5 questions reflecting the urge to smoke due to craving and withdrawal symptoms, which are considered essential for addiction.

c Measured by 3 questions capturing decreased sensitivity to nicotine or an increasing number of cigarettes smoked per day since smoking onset.

d Measured by 3 items referring to the constancy of smoking behavior without interruption.

e Measured by 2 items representing the degree to which smoking was prioritized over other behaviors.

f Measured by 4 items indicating the development of day-to-day patterns of smoking and increasing resistance to change.

Chi-square tests indicated significant differences among the 5 smoking status groups on each of the sociodemographic characteristics. Compared with very light smokers, never smokers were more likely to be young (aged 18–20), while former, light, and heavier smokers were more likely to be older. Compared with very light smokers, never smokers were more likely to be black and light or heavier smokers were less likely to be black; compared with very light smokers, never smokers and former smokers were more likely to be Hispanic and light or heavier smokers were less likely to be Hispanic. Compared with very light smokers, never and former smokers were more likely to have at least some college education and never smokers were more likely to be enrolled in school; compared with very light smokers, light or heavier smokers were less likely to have at least some college education and less likely to be currently enrolled in school. Compared with very light smokers, all of the other groups of smokers were more likely to be married.

Never smokers were less likely than very light smokers to report lifetime depression and past month psychological distress, less likely to binge drink or use illicit drugs, and never smokers perceived smoking to be of higher risk (Table 3). Former smokers were less likely than light smokers to have past month psychological distress, less likely to binge drink or use illicit drugs, and they perceived smoking to be of higher risk. Light and heavier smokers did not differ from very light smokers in regards to the likelihood of lifetime depression, past-month psychological distress, or past-month substance use. Light and heavier smokers, in comparison with very light smokers, perceived smoking as lower risk. For all categories, very light smoking was associated with higher risk of past month binge drinking, although the finding was not significant for light and heavier smokers.

**Table 3:** Multinomial Logistic Regression Analysis of Psychosocial Factors Predicting Smoking Status^a^ for the Full Sample of Women Aged 18 to 25 Years, National Survey of Drug Use and Health, 2011 (n = 9,789)

Variable	Never Smokers, OR (95% CI)	Former Smokers, OR (95% CI)	Light Smokers, OR (95% CI)	Heavier Smokers, OR (95% CI)
Past month psychological distress^b^	0.70 (0.56–0.87)	0.74 (0.60–0.92)	1.17 (0.90–1.52)	1.30 (0.91–1.87)
Lifetime depression^c^	0.62 (0.53–0.73)	0.97 (0.83–1.13)	1.09 (0.88–1.35)	0.91 (0.67–1.24)
Past month binge drinking^d^	0.21 (0.18–0.24)	0.47 (0.41–0.54)	0.87 (0.73–1.04)	0.86 (0.66–1.11)
Past month illicit drug use^e^	0.18 (0.15–0.22)	0.45 (0.39–0.53)	1.19 (0.98–1.45)	1.17 (0.89–1.55)
Perceived risk of smoking^f^	1.28 (1.18–1.40)	1.31 (1.20–1.43)	0.78 (0.70–0.87)	0.68 (0.59–0.78)

Abbreviations: CI, confidence interval; OR, odds ratio.

a Very light smokers were current smokers who smoked 5 or fewer cigarettes per day (cpd) within the past 30 days; light smokers smoked 6 to 16 cpd within the past 30 days; and heavier smokers smoked more than 16 cpd within the past 30 days. The reference category is very light smokers. Control variables are age group, ethnicity, education, and marital status (not shown in the table).

b Six symptoms addressing frequency of psychological distress during the past 30 days were measured on a scale ranging from 0 to 4, with responses coded with 0 as none of the time, 1 as a little of the time, 2 as some of the time, 3 as most of the time, and 4 as all of the time. Item scores were summed, with total scores ranging from 0 to 24. Total scores less than 13 were coded as not having past month psychological distress (reference group), and total scores 13 or higher were coded as having psychological distress.

c If participants had at least 5 of 9 symptoms of depression (eg, depressed mood or loss of interest or pleasure in daily activities) lasting at least 2 weeks in a year, they were coded as having a lifetime major depressive episode.

d Defined as drinking 5 or more drinks on the same occasion on at least 1 day in the past 30 days.

e Presence or absence of illicit drug use, determined from questions on use of hallucinogens, heroin, marijuana, cocaine, inhalants, and “any psychotherapeutics” (ie, pain relievers, tranquilizers, stimulants, and sedatives).

f From responses to the question “How much do people risk harming themselves physically and in other ways when they smoke 1 or more packs of cigarettes per day?” Responses were coded with 1 as no risk, 2 as slight risk, 3 as moderate risk, and 4 as great risk.

Light and heavier smokers, in comparison to very light smokers, did not differ in age at initiation of smoking (Table 4). Light and heavier smokers, in comparison to very light smokers, were more likely to be daily smokers and to report higher levels of smoking drive, nicotine tolerance, and rigid smoking patterns (stereotypy). Heavier smokers, in comparison to very light smokers, were more likely to prioritize smoking over other behaviors.

**Table 4:** Multinomial Logistic Regression Analysis of Psychosocial Factors Predicting Smoking Status for Current Smokers^a^ Among Women Aged 18 to 25 Years, National Survey of Drug Use and Health, 2011 (n = 2,941)

Variable	Light Smokers, OR (95% CI)	Heavier Smokers, OR (95% CI)
Starting age	0.97 (0.93–1.01)	0.97 (0.92–1.02)
Daily smoker	4.66 (3.53–6.14)	4.35 (2.76–6.85)
Smoking drive^b^	2.07 (1.79–2.41)	2.10 (1.71–2.59)
Nicotine tolerance^c^	1.13 (1.01–1.28)	1.41 (1.20–1.65)
Continuous smoking^d^	0.89 (0.78–1.01)	1.01 (0.85–1.21)
Behavioral priority^e^	1.07 (0.89–1.27)	1.47 (1.20–1.81)
Stereotypy^f^	1.56 (1.36–1.80)	2.16 (1.77–2.63)

Abbreviations: CI, confidence interval; OR, odds ratio.

a Very light smokers were current smokers who smoked 5 or fewer cigarettes per day (cpd) within the past 30 days; light smokers smoked 6 to 16 cpd within the past 30 days; and heavier smokers smoked more than 16 cpd within the past 30 days. The reference category is very light smokers. Control variables are age group, ethnicity, education, and marital status (not shown in the table).

b Measured by 5 questions reflecting the urge to smoke due to craving and withdrawal symptoms, which are considered essential for addiction.

c Measured by 3 questions capturing decreased sensitivity to nicotine or an increasing number of cigarettes per day since smoking onset.

d Measured by 3 items referring to the constancy of smoking behavior without interruption.

e Measured by 2 items representing the degree to which smoking was prioritized over other behaviors.

f Measured by 4 items indicating the development of day-to-day patterns of smoking and increasing resistance to change.

## Discussion

The purpose of our study was to identify the characteristics of very light smokers compared with nonsmokers and smokers at other levels of consumption among women in emerging adulthood. We found that very light smoking and intermittent smoking were common in this national sample of US women in emerging adulthood. Almost a fifth of all participants and about three-fifths of current smokers were very light smokers, and almost half of current smokers were nondaily smokers. The rate of very light smoking among current smokers in our study of women in emerging adulthood is comparable with other estimates among emerging adults (2,8). Consistent with findings in previous research, we found that women were more likely to engage in very light and intermittent smoking than heavier or daily smoking (4,17), and intermittent smoking was particularly characteristic of very light smokers (18).

The present findings extend previous research on sociodemographic characteristics associated with overall cigarette use (1) to very light smoking among emerging-adult women. Very light smokers were relatively more likely than other smokers to be young (aged 18–20), from a minority group, and to have some college education and relatively less so than never smokers. They were also less likely to be married than any other group. The present results also extend literature showing a link between smoking and both psychological distress and substance misuse (19) to very light smoking among emerging-adult women. Very light smokers showed less favorable psychological and substance use profiles than current nonsmokers. They did not differ from light or heavier smokers on psychological distress, lifetime depression, or misuse of alcohol and illicit drugs. In terms of perceived smoking risk, very light smokers fell between current nonsmokers and individuals who smoked at higher levels. Moreover, very light smokers were more likely to be nondaily, intermittent smokers and less likely to report signs of nicotine dependence than were individuals who smoked at higher levels. Similarly, previous studies found that very light smokers were more likely to plan to quit and to have higher self-efficacy for quitting than light or heavy smokers (20).

Overall, these findings point to a unique profile for very light smokers among women in emerging adulthood. With the exception of their being less likely to be married than all other study groups, very light smokers consistently fell between participants who smoked at higher levels and never smokers in sociodemographic characteristics. Their poorer psychological adjustment and greater tendency to misuse other substances generally contrasted with nonsmokers but were similar to characteristics of participants who smoked at higher levels. However, in terms of characteristics directly linked to smoking, the profile of very light smokers looked more promising for smoking cessation interventions. Compared with individuals who smoked at higher levels, very light smokers recognized higher risks in smoking, were less likely to report signs of nicotine dependence, and were more likely to be nondaily smokers.

These findings also suggest several pathways to very light smoking among women in emerging adulthood. The economic cost of cigarettes may maintain smoking at a very light level among poor women of minority status (21). Social features of college life, including weekend partying, may promote smoking at a very light level among college women (22). Emotional distress and multiple substance misuse may serve to both initiate and maintain very light smoking (23). Perceptions that smoking is of lower risk compared with the perceptions of current nonsmokers, reinforced by perceptions of fewer signs of being dependent on nicotine compared with the perceptions of current light and heavier smokers, may serve to further maintain very light smoking at an attitudinal level. In general, research on smoking behavior among young adults in Australia (22), Canada (23), and Finland (24) shows results similar to those reported here among US women.

The findings from our study have implications for research and practice. Health educators and health care providers working with women in emerging adulthood need to recognize the high prevalence of very light smoking in this population and screen for any level of tobacco use. In addition, smoking cessation interventions should be tailored to the higher likelihood of very light smokers being relatively young, being from minority groups, and having at least some college education compared with other smokers. Further, as with other smokers, interventions with very light smokers need to address issues of psychological maladjustment and substance misuse. Although a lack of identification as smokers (25) may present a barrier, our findings indicated that very light smokers perceive higher risks in smoking than do other smokers and tend to report less nicotine dependence, which may provide an opportunity for intervention. Because women in emerging adulthood are of childbearing age, preventing smoking before or during pregnancy is critical for maternal and child health (11).

Some studies report that the smoking pattern of very light smokers is unstable (24). However, other research suggests that an intermittent pattern of smoking may be maintained for an extended period of time (17,26,27). Although very light smokers express an interest in quitting (20), they may be less motivated to quit (28) and may have relapse rates similar to those of heavy smokers (29). Nondaily smokers may be resistant to quitting because of their reluctance to identify themselves as smokers (25). Young adults have the highest prevalence of quit attempts yet the lowest quit ratio (ie, the ratio of former smokers to ever smokers) among all age groups (1,11). Future longitudinal research should focus on the course of very light smoking in emerging adulthood.

Our study has several limitations. The data were self-reported and are subject to recall bias, common variance due to the same method of data collection, and social desirability of responses. The severity of psychological disorders was not measured in NSDUH 2011 and could not be addressed in this study. Because the data were cross-sectional, we could not address patterns of change among subgroups of very light smokers.

Although any level of smoking is harmful to young women’s health, the prevalence of light smoking has increased among young women (2). Yet, little is known about very light smoking in women in emerging adulthood (3). The tobacco industry developed campaigns to appeal to young women smokers (30). Advertising aimed at women attempts to associate smoking with independence, attractiveness, and sophistication, traits that are especially likely to appeal to young women (9). To meet the challenge of the tobacco industry, smoking intervention programs and policies directed at emerging-adult women need to be based on an understanding of the diverse characteristics — demographic, psychological, behavioral, and attitudinal — associated with very light smoking in this population.
